# Person-centred medicine in the care home setting: development of a complex intervention

**DOI:** 10.1186/s12875-024-02437-x

**Published:** 2024-05-27

**Authors:** Kirsten Høj, Hilary Louise Bekker, Flemming Bro, Anne Estrup Olesen, Jette Kolding Kristensen, Line Due Christensen

**Affiliations:** 1 Research Unit for General Practice, Aarhus, Denmark; 2 Department of Clinical Pharmacology, Aalborg University Hospital, Aalborg, Denmark; 3 Department of Clinical Pharmacology, Aarhus University Hospital, Aarhus, Denmark; 4 Leeds Institute of Health Sciences, University of Leeds, Leeds, UK; 5 Research Centre of Patient Involvement, Aarhus University Hospital, Aarhus, Denmark; 6 Department of Public Health, Aarhus University, Aarhus, Denmark; 7 Department of Clinical Medicine, Aalborg University, Aalborg, Denmark; 8 Centre for General Practice, Aalborg University, Aalborg, Denmark

**Keywords:** Person-centred medicine, Medicines optimisation, Residential facilities, Health services for the aged, Primary health care, Intervention development, Co-production, Denmark

## Abstract

**Background:**

Person-centred medicine is recommended in the care of older patients. Yet, involvement of care home residents and relatives in medication processes remains limited in routine care. Therefore, we aimed to develop a complex intervention focusing on resident and relative involvement and interprofessional communication to support person-centred medicine in the care home setting.

**Methods:**

The development took place from October 2021 to March 2022 in the Municipality of Aarhus, Denmark. The study followed the Medical Research Council guidance on complex intervention development using a combination of theoretical, evidence-based, and partnership approaches. The patient involvement tool, the PREparation of Patients for Active Involvement in medication Review (PREPAIR), was included in a preliminary intervention model. Study activities included developing programme theory, engaging stakeholders, and exploring key uncertainties through interviews, co-producing workshops, and testing with end-users to develop the intervention and an implementation strategy. The Consolidated Framework for Implementation Research and the Interprofessional Shared Decision Making Model were used. Data were analysed using a rapid analysis approach.

**Results:**

Before the workshops, six residents and four relatives were interviewed. Based on their feedback, PREPAIR was modified to the PREPAIR *care home* to fit the care home population. In total, ten persons participated in the co-producing workshops, including health care professionals and municipal managerial and quality improvement staff. The developed intervention prototype was tested for three residents and subsequently refined to the final intervention, including two fixed components (PREPAIR *care home* and an interprofessional medication communication template) delivered in a flexible three-stage workflow. Additionally, a multi-component implementation strategy was formed. In line with the developed programme theory, the intervention supported health care professionals´ awareness about resident and relative involvement. It provided a structure for involvement, empowered the residents to speak, and brought new insights through dialogue, thereby supporting involvement in medication-related decisions. The final intervention was perceived to be relevant, acceptable, and feasible in the care home setting.

**Conclusion:**

Our results indicate that the final intervention may be a viable approach to facilitate person-centred medicine through resident and relative involvement. This will be further explored in a planned feasibility study.

**Supplementary Information:**

The online version contains supplementary material available at 10.1186/s12875-024-02437-x.

## Background

Care home residents are often exposed to complex medication regimens and excessive polypharmacy due to a high prevalence of multimorbidity and symptoms [1, 2]. Polypharmacy increases the risk of potentially inappropriate medication which occurs when the risk of harms exceeds the expected benefits of treatment [3]. Potentially inappropriate medication is observed in almost half of care home residents [4] and may lead to reduced quality of life, hospital admission, and premature death due to adverse drug events [5].

A person-centred approach to medication-related decisions is recommended as a guiding principle in the care of older patients with multimorbidity [6, 7]. Person-centred care is defined as care that is guided by an individual’s preferences, needs, and values [8]. In the care home population, medication-related decisions are highly preference-sensitive, as the existing evidence base is typically based on younger populations with fewer health conditions, which may not apply to older patients [4, 9, 10]. Incorporating individual preferences and priorities into medical decision-making can improve treatment adherence, patient satisfaction, and perceived well-being and quality of life [11–18]. However, research indicates that involvement of residents and their relatives in medicines remains limited in clinical practice [19–23].

Important barriers to resident and relative involvement in medication-related decisions are the awareness and attitudes of health care professionals (HCPs) [24]. HCPs often perceive care home residents as not being capable of or not wanting to be involved in their care [25]. Similarly, HCPs sometimes believe that the relatives do not want to be involved, or they perceive relative involvement to be time-consuming, not helpful, and sometimes problematic [26]. Research indicates that residents and their relatives generally want to be involved [27–29], but many residents are reticent, because they think that their HCPs are not receptive to their perspectives, or they do not know how to be involved [30]. Recent reviews have explored tools for practicable elicitation of patient preferences in the context of geriatric polypharmacy [31]. However, so far, no ideal method has been identified to support patients, relatives, and HCPs in this task, and the need for clinically applicable strategies has been stressed [31].

Recently, a new patient involvement tool, the PREparation of Patients for Active Involvement in medication Review (PREPAIR), was developed with the aim to encourage the involvement of patients with polypharmacy in medicines optimisation in general practice [32]. PREPAIR is a simple, five-item questionnaire with a three-point Likert scale response-option. The five items deal with 1) adverse drug reactions, 2) excess medication, 3) unnecessary medication, 4) medication satisfaction, and 5) an open-ended item on medication-related topics for discussion. PREPAIR is completed by the patient as preparation before consulting the GP at the yearly chronic care consultation. During the consultation, PREPAIR supports a person-centred dialogue about the patient´s medication. PREPAIR was demonstrated as a feasible instrument to facilitate patient involvement in this setting. Based on these findings, we hypothesised that PREPAIR could be useful in the care home setting to facilitate involvement of residents and their relatives in medication-related decisions and thereby support person-centred medicine. However, although PREPAIR itself may be simple, making it useful and integrated into the care home setting requires changing the reasoning and actions of multiple stakeholders such as residents, relatives, and HCPs. Furthermore, it requires interprofessional coordination and communication.

Changing professional behaviour is challenging, and interventions aiming to do so are often complex [33, 34]. Interventions can be considered complex if they contain multiple interacting components; they require new behaviours of those delivering and receiving the intervention; and/or there is a need to tailor the intervention to different contexts and settings [33]. The Medical Research Council (MRC) guidance for developing and evaluating complex interventions divides the research process into four phases: development or identification of the intervention, feasibility, evaluation, and implementation [33]. Complex interventions require thorough development to enhance the chance of being effective and widely adopted into routine care. Development refers to the whole process of designing and planning an intervention, and a key source can be an existing intervention that has the potential of being adapted to a new population or setting [33].

### Aim

The present study is the first part of a larger project with the overall aim to develop (MRC phase 1) and feasibility test (MRC phase 2) a complex intervention to support person-centred medicine in the care home setting through resident and relative involvement and interprofessional communication. In this paper, we report on the MRC phase 1, the development phase.

The specific research aims in the development phase were to:

1. develop a complex intervention and implementation strategy in a co-producing process.
2. test and refine the complex intervention as part of the development process.

## Methods

### Study design

**Fig. 1:**
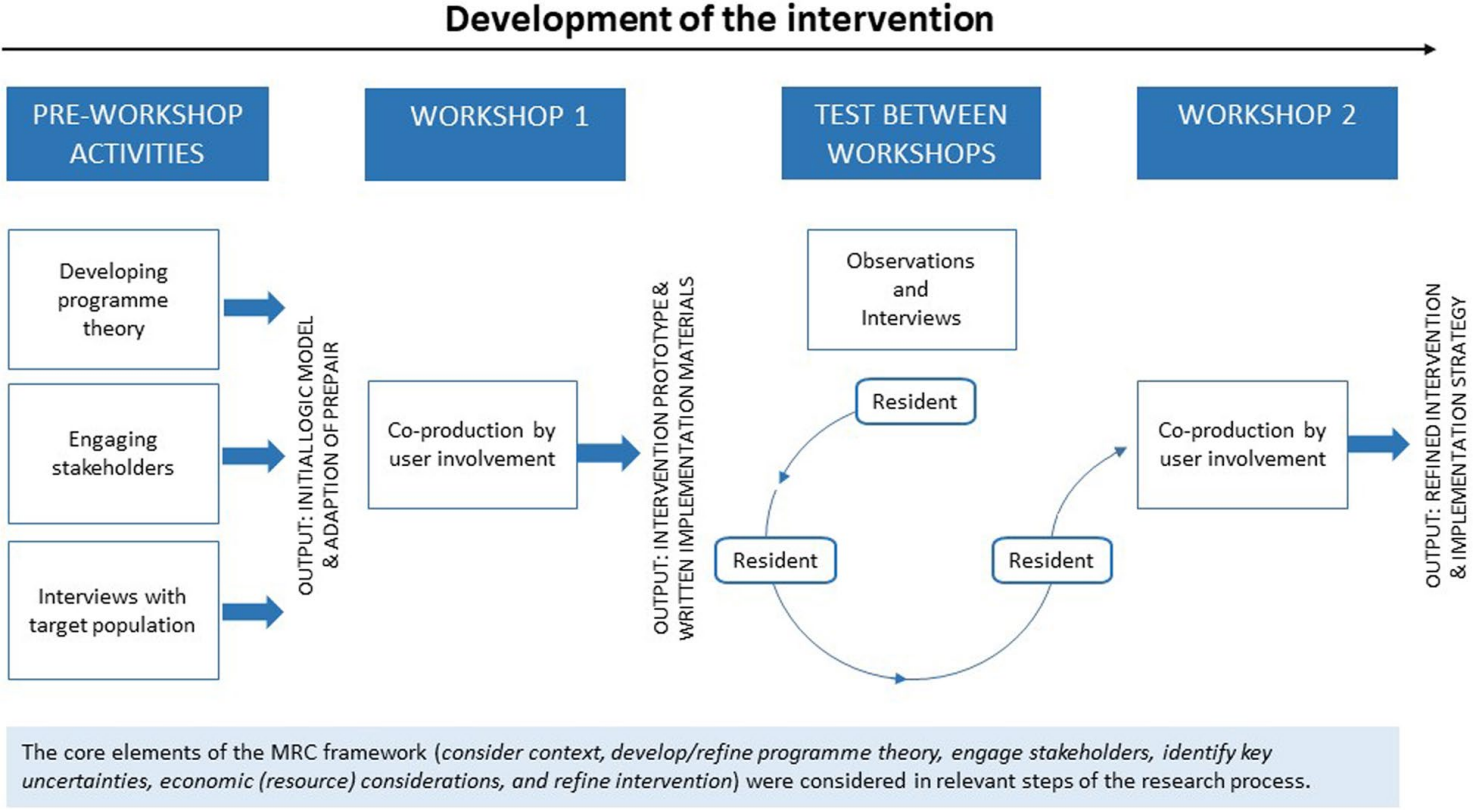
Overview of study activities and their outputs, including specification of the core elements from the Medical Research Council (MRC) framework [33] considered in the research process

The overall approach in this study was based on the MRC guidance [33, 35]. In each MRC phase, a set of core elements should be considered. In this study, several activities were undertaken, through which the core elements were considered (Fig. 1). The research design involved elements from coproduction [36] and rapid cycle [37] research. In this approach, researchers and end-users collaborate in an iterative process based on interdisciplinary collaboration and rapid qualitative analysis with an ongoing exchange between research and practice.

The study was conducted in close collaboration with the Municipality of Aarhus, Denmark. Furthermore, it was endorsed by the Danish Society for Patient Safety [38] and the municipal Organization of General Practitioners, Central Denmark Region [39]. The development phase took place from September 2021 to March 2022. The present paper conforms to the GUIDance for the rEporting of intervention Development (GUIDED) [40].

### Context

Understanding the context within which the intervention needs to be integrated is essential to explore the mechanisms enabling the implementation of an intervention in usual practices [33]. According to Danish law, elderly, frail citizens are suitable for care home residency, if they need all-day care. The allocation of residency is made by the municipalities [41]. The care for the residents is provided mainly by a team of nurses, social and health care assistants (SoHAs), and social and health care helpers.

In 2017, the designated GP model was introduced in Danish care homes [42]. In this model, one or several GPs with private clinics are assigned to serve as designated GP in a care home. Care home residents can keep their regular GP when moving into a care home, but new residents are encouraged to register with the designated GP. The model has been implemented in all care homes in the Municipality of Aarhus; however, the role of the designated GP is still in development. In Denmark, GPs operate as independent contractors that provide primary medical care under a collective agreement with the Danish Regions [43]. The GPs are responsible for most prescription medications [44] and chronic care management [45]. Primary care services are mainly tax-financed and free of charge for patients.

### Target population

A priori, newly arrived residents and their relatives were considered to be a relevant target group for the intervention. When elderly citizens move to a care home facility in Denmark, the majority chose to be assigned to the designated GP [46]. In connection with this shift, it is common that the residents use of medicine is reassessed in terms of potential risks and beneficial effects. However, as the designed GP typically has no prior relation to a new resident, the needs, preferences, and values of the resident concerning their medication will need to be explored to ensure person-centred medicine. Thus, newly arrived residents at the participating care homes were considered eligible if they chose to register with the designated GP. The single exclusion criteria for the residents was severe cognitive impairment as judged by the HCPs. Care home residents are a heterogeneous group with different health conditions, and many suffer from cognitive impairment. A way of supporting cognitively impaired residents in being involved is through the involvement of relatives who know the resident and can speak on their behalf [47, 48]. Therefore, relatives were included in the target group as well.

### Developing programme theory

**Fig. 2:**
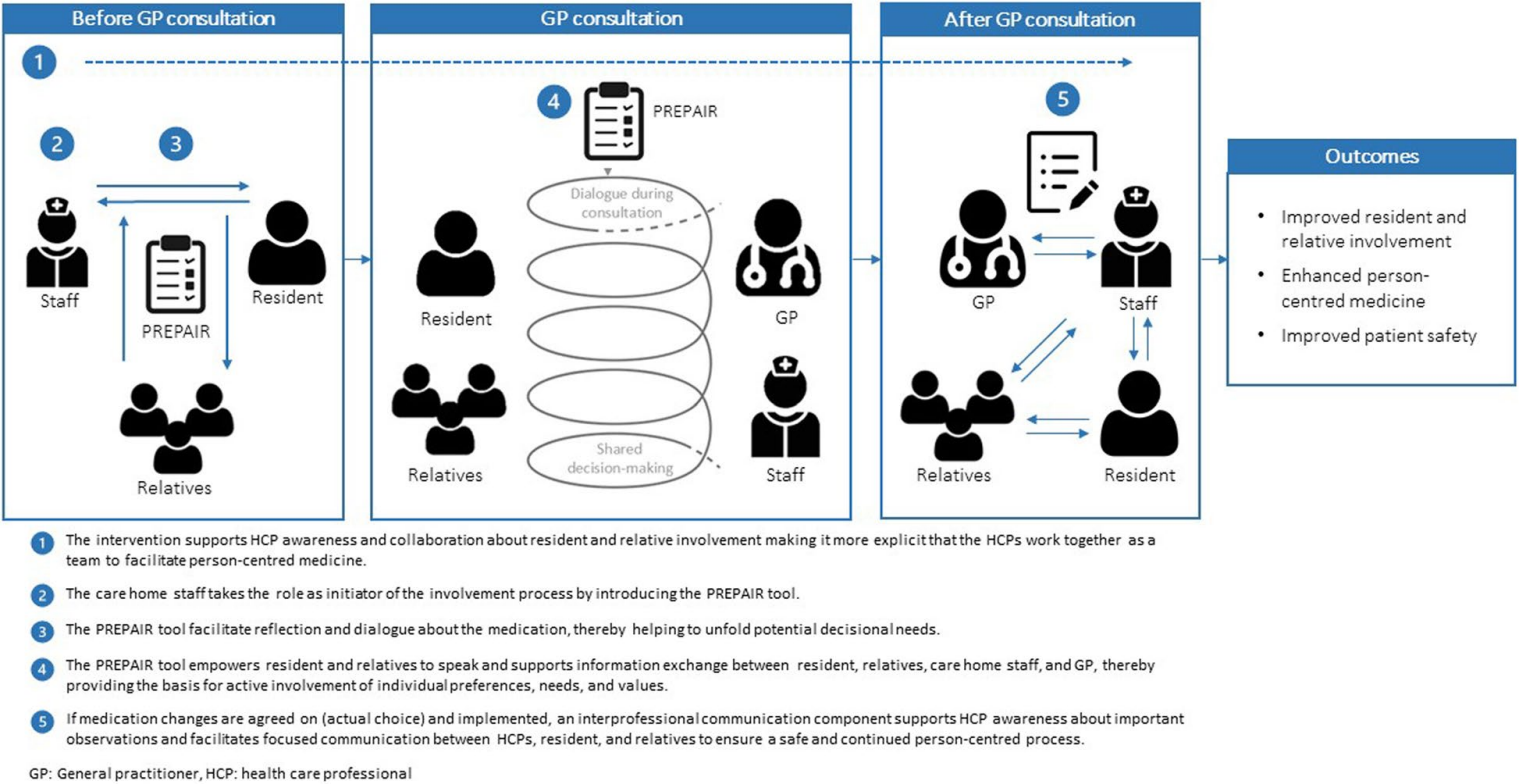
Linear logic model of the intervention. Modified from Sandbæk et al. [32]

A programme theory depicts how an intervention is expected to lead to its effects and under what conditions [33]. Our initial programme theory was inspired by the original PREPAIR study [32] and the Interprofessional Shared Decision Making (IP-SDM) model [49]. The IP-SDM model extends shared decision making to include family members and other caregivers as well as the whole team of HCPs in a person-centred process. Based on the IP-SDM model, relatives in this study were defined as family, surrogate or significant others. The IP-SDM model was inspirational in terms of outlining the key actors and their respective roles in supporting person-centred medicine in the complex care home setting. Figure 2 presents the initial linear logic model of the intervention, the proposed mechanisms of actions, and the expected outcomes. The preliminary intervention model included two key components: a) PREPAIR and b) an interprofessional communication component. These components were to be delivered in a three-stage workflow (before, during, and after the GP consultation).

An additional focus of our programme theory was how to support intervention implementation in the care home setting. Guided by the Consolidated Framework for Implementation Research (CFIR) [50] and literature [51, 52], we developed an initial idea bank of applicable implementation strategies (i.e., methods or techniques used to improve adoption, implementation, sustainment, and scale-up of interventions [52] (Table 1). The CFIR is a comprehensive implementation determinant framework designed to guide a systematic assessment of potential barriers and facilitators that influence implementation outcomes. For the idea bank, we focused on the most relevant construct in the domains Inner Setting, Characteristics of Individuals, and Process. The domains Intervention Characteristics and Outer Setting were considered through stakeholder engaging activities described below.

**Table 1:** Initial idea bank of implementation strategies linked to CFIR domains and the final implementation strategy

Initial idea bank of implementation strategies	Targeted CFIR domain (constructs)	Final implementation strategy
Start-up meeting with the care home manager, selected staff, and the designated GP	• Inner Setting (*Leadership Engagement, Readiness for Implementation*) • Process (*Planning, Engaging, Opinion Leaders*)	Included
Written information materials (e.g., project leaflet, intervention manual)	• Inner Setting (*Access to Knowledge & Information*) • Characteristics of Individuals (*Knowledge & Beliefs about the Intervention, Self-efficacy)*	Included
Intervention instruction video	• Inner Setting (*Access to Knowledge & Information*) • Characteristics of Individuals (*Knowledge & Beliefs about the Intervention, Self-efficacy)*	Not included
Introduction/educational meeting with the entire care home staff group	• Inner Setting (*Access to Knowledge & Information, Readiness for Implementation, Implementation Climate*) • Characteristics of individuals *(Knowledge & Beliefs about the Intervention, Self-efficacy)* • Process (*Engaging*)	Included
Appointed coordinators at each care home	• Process (*Opinion Leaders, Formally Appointed Internal Implementation Leaders, Executing)*	Included
Opportunity for contact with a researcher (telephone, e-mail)	• Process (*Executing*)	Included

*CFIR* Consolidated Framework for Implementation Research, *GP* General practitioner

### Engaging stakeholders

Several activities were performed to engage stakeholders and ensure project progress throughout the development phase. Table 2 provides a description of the stakeholders and their respective roles. Initially, a steering group with representatives from the managerial level of the involved stakeholders was established to support and maintain engagement and to provide inputs and approval at key time points (e.g., approval of the programme theory and the final intervention). Additionally, an operational coordinator group was established, including municipal managerial and quality improvement staff and the project leaders, the authors KH and LDC. Ad hoc meetings were held in the coordinator group to resolve practical issues (e.g., concerning recruitment) and discuss project progress. Other stakeholder involving activities included preworkshop interviews with residents and relatives, co-production workshops, and testing between workshops which will be described in the following.

**Table 2:** Description and role of stakeholders

	Stakeholder	Description	Role*
Managerial level	Municipal management	Municipal management of the Department of Care for the Elderly and Department of Quality and Safety	• Steering group • Operational coordinator group
	Municipal Organisation of GPs	The municipal Organisation of GPs is a group of GPs in a municipality who represent the GPs in the municipal medical collaboration, including appointing representatives to a Municipal Medical Committee	• Steering group
	Danish Society for Patient Safety	Danish Society for Patient Safety is an independent organization working to improve patient safety across Danish healthcare. Danish Society for Patient Safety is mainly funded by support from the Danish regions and Local Government Denmark	• Steering group
	Resident and relative representative	A professional resident and relative representative appointed by the Danish Society for Patient Safety	• Steering group
	Municipal quality improvement staff	Municipal staff with special education in quality improvement (e.g., Master of Science in Public Health or 2 years of further education on top of a Bachelor of Nursing degree)	• Operational coordinator group • Co-production workshop
	Care home manager	Local manager at the care home	• Co-production workshops
	Researchers	A multidisciplinary research group with expertise within the fields of intervention development, primary care, patient involvement, and clinical pharmacology	• Steering group • Operational coordinator group • Co-production workshops • Testing between workshops • Data collection and analyses
	Municipal management	Municipal management of the Department of Care for the Elderly and Disabled and Unit of Quality and Innovation	• Steering group • Operational coordinator group
User level	Resident	An older person living at the care home	• Preworkshop interviews • Testing between workshops
	Relative	A family member or personal carer for the resident	• Preworkshop interviews • Testing between workshops
	Care home staff	Care home staff include care home nurses (42 month of basic education), social and health assistants (32 month of basic education), and social and health helpers (14 month of basic education)	• Co-production workshops • Testing between workshops
	Designated GP	A GP who is dedicated to a specific care home	• Steering group • Co-production workshops • Testing between workshops

*GP* General practitioner

^*^The described roles were represented by different persons, with the exception of one designated GP that was represented in both the steering group and the co-production workshops

### Preworkshop interviews with residents and relatives

A key uncertainty was whether the care home residents would be able to meaningfully interact with the PREPAIR tool and find it useful. Therefore, preworkshop interviews were undertaken to explore residents' and relatives’ views on the acceptability and feasibility of PREPAIR and to explore their experiences and preferences for involvement in medication-related decisions. The interviews were conducted from September to October 2021. Residents and relatives from one rural and two urban care homes were recruited by convenient sample. Semi-structured, audio-taped interviews were performed by LDC at the care homes in the residents’ living rooms. The interview guide was inspired by a similar study on older adults about attitudes towards medications [29] (Supplementary Material 1).

### Co-production workshop 1

The first co-production workshop was conducted in November 2021. All participants (Table 3) were purposively recruited in collaboration with the municipality. The workshop lasted three hours and comprised three sessions. In the first session, the prespecified theme `resident and relative involvement´ was unfolded. The second session dealt with the prespecified theme `interprofessional communication´. In these two sessions, individual and group activities were performed, through which barriers and facilitators concerning the prespecified themes in the context of the intervention were identified and discussed based on insights from the preworkshop interviews, evidence-based knowledge, and practice-based experiences. In the final session, a structured consensus process was used to agree on the intervention content (i.e., *what* will be delivered) and intervention delivery (i.e., *how* it will be delivered) of the intervention prototype. The workshop was facilitated by KH and LDC and included the use of post-its and flipboard notes during the processes. Furthermore, the processes were audio-taped and video-recorded.

**Table 3:** Overview of participants across study activities

	Phase 1: Development of the intervention			
	Pre-workshop	Workshop 1	Between workshop testing	Workshop 2
	Interviews	Co-production	Interviews	Co-production
**Care home 1**	I. RS1, RL1, RL2 II. RS2, RL3			
**Care home 2**	I. RS3, RL4 II. RS4	GP1, N1, SoHA1	I. RS7	GP1, CM2, N1*
**Care home 3**	I. RS5 II. RS6	GP2, SoHA2, SoHA3, CHM1	I. RS8 II. RS9	GP2, CM1, SoHA2*
**Municipality**		DM1, QS1		

*RS* Resident, *RL* Relative, *GP* General practitioner, *N* Nurse, *SoHA* Social and health care assistant, *CM* Care home manager, *DM* District manager, *QS* Quality improvement staff

^*^Interviews were conducted shortly after the workshop

### Testing the intervention prototype

The intervention prototype and first draft of written implementation materials were tested from December 2021 to February 2022. A priori, we had planned to include six residents and their relatives (three in each care home). However, due to the Covid-19 situation and a limited flow of new residents moving in during the testing period, we were able to recruit only three residents, of which none were newly arrived, and no relatives.

During testing, observations and interviews were performed with a focus on exploring key uncertainties concerning the delivery, acceptability, and feasibility of the intervention. Observations of the first two intervention stages (staff-led conversation and GP consultation) were undertaken. We used a complete observer approach, where the researcher observed without participation [53]. The observation protocol was focused on how the intervention was performed and received. During observations, descriptive and reflexive notes were made. Semi-structured, audio-taped interviews with residents and relatives were conducted shortly after the testing. The interview guide was inspired by questions related to patient involvement which had been adapted to and validated in a Danish setting [54] (Supplementary Material 2). The interviews and the observations were conducted by LDC at the care homes.

### Co-production workshop 2

The second workshop was conducted in February 2022 and was planned to include the same group of participants as in workshop 1. However, due to busyness at the care homes and acute illness, only four participants attended (Table 3). The workshop lasted three hours and comprised to sessions with group activities and general discussion. The first session dealt with positive and challenging experiences of the intervention based on the testing and, subsequently, intervention refinement. The second session focused on further development of the implementation strategy. The workshop was facilitated by KH and LDC. The nurse and SoHA that were unable to attend due to acute illness were interviewed individually by telephone after the workshop by KH and LDC. As in the workshop, the audio-taped interviews focused on the informants´ experiences with the intervention and their thoughts about the implementation strategy.

### Data analyses and synthesis

Overall, data analyses were based on a rapid analysis approach. We were inspired by the approach described by Neal et al. [37], in which prespecified key research foci are identified directly from audio recordings, thereby eliminating the need to have time-consuming verbatim transcriptions and line-by-line coding while still capturing essential information and allowing for new themes to emerge. The key research foci in each step (preworkshop interviews, co-producing workshop, and testing) have been described in the respective sections above. The results from each step were presented and discussed ad hoc at meetings with members of the research team.

For the audio recordings from the preworkshop interviews, the analysing approach was to identify predetermined research foci as well as new emerging themes. Following the first initial coding by LDC, meaningful data units were organised into sub-themes, which were then categorised under overarching themes. Hereafter, LDC and KH collaboratively refined the sub-themes and overarching themes. Exemplifying citations were transcribed. The results from the preworkshop interviews and subsequent discussions in the research team led to adaption of the PREPAIR tool to fit the care home setting. The adapted tool (PREPAIR-CH) and results from the preworkshop interviews were presented in workshop 1.

During workshop 1, the data analysis was done in co-producing processes with the workshop participants. Post-it notes produced through group activities in the first two sessions were organised into barriers and facilitators under the prespecified themes. In the final session, the emerged sub-themes were reevaluated systematically in relation to the proposed preliminary intervention model through general discussion. In this structured consensus process, existing and new ideas of intervention components and delivery methods were either accepted or rejected in agreement between the workshop participants and the researchers. LDC and KH subsequently drafted the intervention prototype and first written implementation materials. The intervention prototype was presented to and approved by the entire research team. Post hoc, exemplifying quotes were identified in the video- and audio materials.

In the analysis of audio recordings and field notes from the testing, the same analysing approach as described for the preworkshop interviews was used. The findings were discussed by researchers (LDC, KH, FB) and with the municipal managerial level prior to presentation in workshop 2.

Finally, during workshop 2, the data analysis was again done in co-producing processes with the workshop participants. Post-it notes produced through group activities in the first session were categorised into positive and challenging intervention experiences. The emerging subthemes guided the subsequent process of agreeing on intervention refinements through general discussion. In the second session, the workshop participants discussed elements for the implementation strategy in groups based on the initial idea bank (Table 1). Hereafter, the elements were pragmatically categorised into relevant/important or rejected through general discussion between the workshop participants and the researchers (KH, LDC). The elements agreed on in the workshop were included in the final implementation strategy. The final intervention was approved by the whole research team and the steering committee. Post hoc, exemplifying quotes were identified in the video- and audio materials.

## Results

### Pre-workshop interviews with residents and relatives

In total, six interviews were conducted, including 6 residents of which 3 were accompanied by relatives (Table 3). They uncovered different attitudes and preferences regarding involvement in medication-related decisions. Furthermore, feedback on PREPAIR was provided, which contributed to adaption of the tool.

#### Attitudes and preferences toward involvement in medication-related decisions

All residents expressed a desire to be involved in their medication. Some wished to be informed; others had actively tried to influence their medication or be in some kind of control. For instance, a resident counted the pills to ensure she got the right treatment. Another resident had requested to have a specific medication deprescribed. The resident said:

*”I had some diuretic… but I’ve stopped that. I simply asked to be exempted from that because I don’t need it any longer”* (RS4, care home 2).

However, most residents felt that their knowledge about the medication was limited and did not know how to be involved in decisions about their medication. The residents had the impression that the HCPs made the decisions about their medications. Nonetheless, the residents and their relatives trusted the HCPs and believed that they made the right decisions.

The relatives also expressed that they would like to be involved in the residents' medication. Some relatives put much effort into being involved. A relative said:

*”We have discussed it quite a bit with the nurse and her [the resident’s] contact person because we obviously don’t want my mother to get more medications than what is good.” (*RL1, care home 1*).*

Another relative stated that she wished to be more involved. Primarily, because she wanted to be able to talk to the resident about the medication, but also because she had encountered problems with the medication at the care home. A resident expressed a desire for more involvement from relatives. Oppositely, not all residents wanted the relatives to be involved.

Thus, both residents and relatives wanted to be involved in medication-related decisions, although some variation was observed in terms of the preferred level of involvement and the role of relatives.

#### PREPAIR feedback

Overall, the residents and their relatives found PREPAIR useful as a dialogue tool to support a conversation about the medication. A relative stated:

“*It can structure a conversation so that you get to touch upon topics that you would otherwise not have discussed*” (RL3, care home 1).

When looking more into the details of the layout and content of PREPAIR, several areas for improvement were pointed out by the residents and their relatives: the font was too small; the response categories were tricky to answer; and it was difficult to distinguish between two of the statements (i.e., statements three and four). Additionally, it was noted that most residents would not be able to fill out the form by themselves.

#### Adaption of PREPAIR

**Fig. 3:**
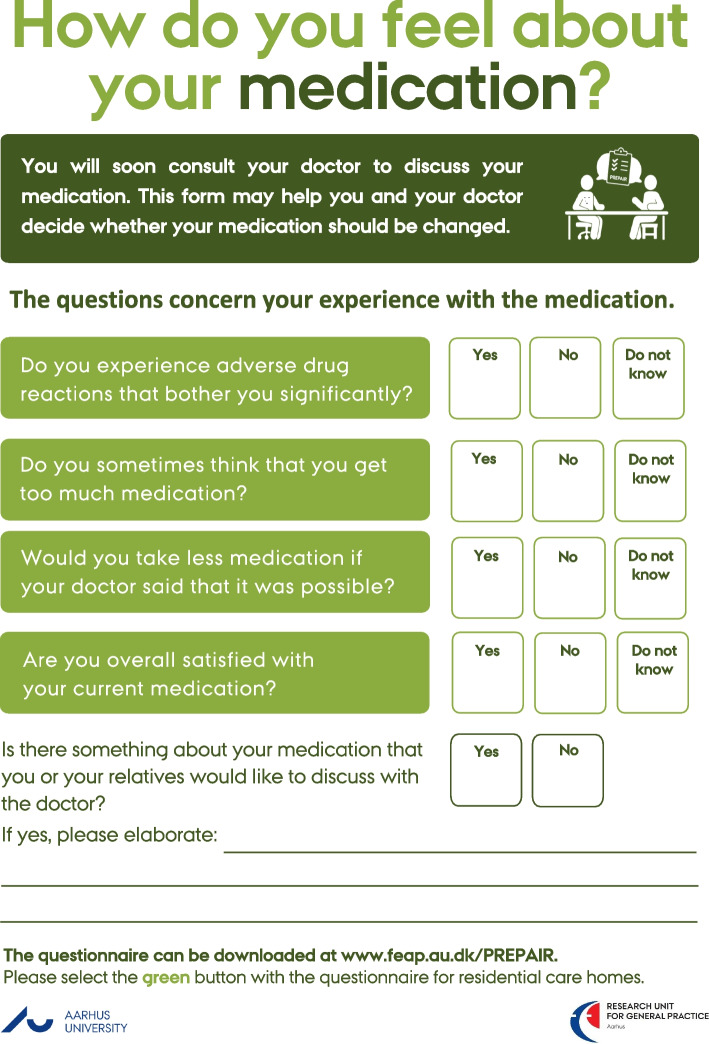
PREPAIR care home

Based on the resident and relative feedback and research group discussions, adjustments were made to the original PREPAIR tool: the introductory text was modified, a larger font size was applied; statements were rephrased to questions; and the response categories were revised from a 3-point Likert Scale to the categories yes/no/do not know. Additionally, statement four was replaced with the question “Would you take less medication if your doctor said that it was possible?” inspired by the rPATD [55]. The purpose of the new question was to encourage discussions on deprescribing between residents, relatives, and HCPs. The modified version of PREPAIR was titled: PREPAIR care home (hereafter PREPAIR-CH) (Fig. 3).

### Co-production workshop 1

In the first co-production workshop, various barriers and facilitators related to the prespecified themes `resident and relative involvement´ and `interprofessional communication´ in the context of the intervention were identified. During the final consensus process, the intervention components and delivery methods were discussed and agreed upon.

#### Session 1: barriers to and facilitators of resident and relative involvement

Important barriers to resident and relative involvement were that it takes time and needs to be prioritized to occur more systematically than in the current clinical practice. Varying preferences for involvement among residents and relatives (and sometimes unrealistic expectations among relatives) were also considered as barriers to successful involvement. Additionally, the functional level of the resident was perceived as a barrier, as care home residents have varying degrees of physical (e.g. hearing loss) and cognitive impairments. Among both care home staff and GPs, some doubt existed as to whether it would be possible for all residents to take part in the intervention (e.g. filling out the PREPAIR-CH). A nurse explained:

”*At the care homes where we are, it is probably only a few who will be able to cooperate about such a dialogue tool because their cognition is so bad*.” (N1, care home 2).

All workshop participants recognized that resident and relative involvement was important and found it to be in alignment with the goals and values in the municipality and the care homes. Further, key facilitators of involvement included awareness about involvement among HCPs and using a systematic workflow adapted to the existing practices. Furthermore, early dialogue and alignment of expectations for involvement were found to be potential facilitators of involvement. Additionally, video-consultation was suggested as a way to facilitate involvement of relatives.

#### Session 2: barriers to and facilitators of interprofessional communication

The workshop participants were generally very positive about the existing interprofessional communication about medication and did not articulate any major barriers. They stated that this area had improved considerably since the introduction of the new dedicated GP model in 2017 and highlighted trust and knowledge about each other's professional competencies as fundamental facilitators of successful interprofessional communication. A GP said:

“We have a mutual trust that we do not exploit… well, we do show up after all, we prioritize them (the care home) when there is something that cannot wait until the next visit.” (GP1, care home 2)

Although the communication was already considered to be good, the HCPs suggested that a fixed structure for the communication on medication changes might be a potential facilitator of more relevant and precise communication (“It is also about having a fixed structure” [GP1, care home 2]).

#### Session 3: consensus process

During the final consensus process, the proposed logic model of the intervention was found to fit well with the existing practices and the ideas of the workshop participants. A systematic workflow including a start-up meeting with the resident and relatives was already established for newly arrived residents. This start-up meeting was found to be a good time point to introduce the PREPAIR-CH and fitted well with the idea of early dialog and alignment of expectations for involvement in medication-related decisions. A SoHA said:

*”[with] a new resident […] you capture some things, actually, which mean that we can follow up in due time. There is a good dialogue, and we capture some good things in relation to the medication” (SoHA1, care home 2).*

Additionally, the PREPAIR-CH was found to be a facilitator of HCP awareness and action toward resident and relative involvement. A nurse said:

*“I believe it’s a really good tool – also for us who work in care homes, because it helps increase the focus on, [and to] be curious about, what it is about, what kind of resident we have.” (N1, care home 2).*

Thus, the PREPAIR-CH was agreed on as a key intervention component. Furthermore, an alignment of expectations for involvement in medication-related decisions with residents and relatives was proposed by the staff as an additional element in the start-up meeting to accommodate the variation in attitudes and preferences for involvement across residents and relatives. The notion of video-consultations with relatives was found relevant, but not feasible in clinical practice at this stage, and the idea was rejected. In terms of interprofessional communication, the workshop participants agreed to include a fixed template to communicate medication changes as an element for the intervention to support relevant and precise communication.

#### The intervention prototype

The workshop processes led to the drafting of the intervention prototype and initial implementation materials. As proposed in the programme theory, the intervention prototype was to be delivered by the HCPs in a three-stage workflow:

- Stage 1 was a staff-led conversation about medication as part of the existing start-up meeting for newly arrived residents. The conversation included two elements: a) an alignment of expectations with the resident and relatives involving a clarifying conversation about their expectations and preferences for involvement in medication-related decisions and b) completion of the PREPAIR-CH with the resident and, if possible, the relatives.
- Stage 2 was the GP consultation. When discussing the medication with the resident, the GP would take a starting-point in the completed PREPAIR-CH and the documented alignment of expectations from stage 1. Staff and relatives would participate and facilitate the conversation if desired by the resident.
- Stage 3 included follow-up after the GP consultation, where a medication communication template would be filled out by the GP and sent to the staff as part of the total treatment plan.

The medication communication template was developed after workshop 1 based on insights from the workshop and clinical knowledge in the research team. It included fixed points regarding medication changes and instructions on important observations (Table 4).

**Table 4:**
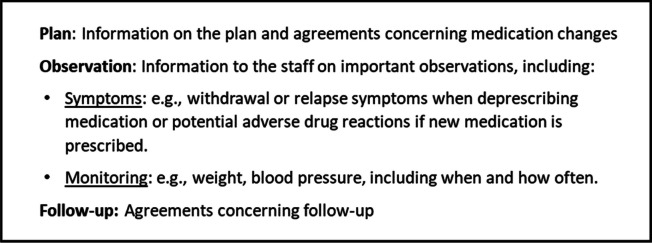
The medication communication template

### Testing the intervention prototype

Three residents tested the intervention and were interviewed (Table 3). Additionally, one staff-led conversation and two GP consultations were observed. The observations revealed some discrepancy between the intended and actual intervention delivery, but, overall, the intervention was found to be acceptable and feasible from the perspective of the residents.

#### Discrepancy between intended and observed intervention delivery

The staff-led conversations were not delivered as part of the start-up meeting as intended due to the lack of opportunity to include newly arrived residents. Instead, the staff-led conversation was delivered to residents that were already settled at the discretion of the staff. The observation of the staff- led conversation revealed that the alignment of preferences was not performed.

#### Resident perspective: acceptability and feasibility

The residents were overall positive about the intervention. A resident stated: *“I think it’s nice that there are some [people] who are interested in you”* (RS7, care home 2). Observations showed that completion of the PREPAIR-CH was led by the staff, who read the form out-loud to the resident. The residents were able to answer the questions, and they all added notes in the open-ended question on topics to discuss with the GP. During the GP consultations, the PREPAIR-CH was actively used in the resident-GP conversation about medication with support from the staff. All residents found the PREPAIR-CH acceptable and feasible to use.

### Co-production workshop 2

In workshop 2, positive and challenging experiences of the intervention from the HCP perspective were brought to light in session 1. The identified challenges led to further refinement of the intervention. Additionally, in session 2, elements of the implementation strategy were discussed and agreed upon.

#### Session 1: positive and challenging intervention experiences

Several positive intervention experiences were articulated. These included that the intervention was found meaningful and in alignment with existing organisational goals; that it provided awareness and structure to support involvement; and that it was feasible within existing working routines and resources.

First of all, the intervention was found to “make sense” and support existing goals in the care homes, which promoted intervention acceptability among HCPs and care home managers. A GP stated:

*”[It] makes good sense to focus more on involvement […] to engage the relatives more*.” (GP1, care home 2).

Furthermore, compared to usual practice, the intervention was perceived to provide additional value in several ways. The intervention was found to increase HCP awareness about resident and relative involvement and to disrupt the habits of usual practice, where the GP and the staff often made decisions about the medication without the resident. A nurse said:

*”The tool invites us to include the patient perspective more, because otherwise this would be something that the GP and I would deal with, but also involving [the resident], I actually find that really good”* (N1, care home 2).

Moreover, the intervention was found to provide a structure that facilitated dialogue and brought new insights into the residents’ believes and preferences regarding their medication for both staff and GPs. A SoHA stated:

*”I don’t believe that I would have captured it [a medication-related issue] if we had not… like… gone into it very specifically in this way because of the tool. (*SoHA2, care home 3*).*

As indicated by the quote, the staff discovered that the use of the PREPAIR-CH revealed medication-related issues that might not have been addressed otherwise. Furthermore, according to the staff, completion of the PREPAIR-CH took about ten minutes on average, which was not perceived as stressful or burdening. A nurse said:

“*When you get sharper on what it’s about, I believe that it will not take a strong presence.*” (N1, CH2).

The staff also perceived the medication communication template to be helpful. Only simple medication changes had been made during testing. However, the nurse stated:

“*It could have been something more specific or something more complicated. It could also be of help to me.*” (N1, care home 2).

Consistent with the staff, the intervention was not perceived as “extra work” by the GPs (”*Medically, it’s not a major extra thing that we are dealing with here" [GP1, care home 2]).* Thus, all HCPs considered the PREPAIR-CH and medication communication template to be feasible within the existing working routines and resources.

The main challenges during testing related to the staff-led conversation and the chosen target group of newly arrived residents and their relatives. Specifically, the alignment of expectations planned to take place as part of the staff-led conversation was perceived as challenging by the staff, and this element was not performed during testing. Furthermore, it proved to be a challenge to include newly arrived residents due to a small turn-over of resident in the testing period. In addition, after having tried the PREPAIR-CH during testing, the staff felt that it might not be appropriate to introduce the PREPAIR-CH in the start-up meeting for newly arrived residents after all. Many issues were on the agenda in the start-up meeting as well as in the first meeting with the designated GP. A SoHA said:

“*There are many things [to discuss] when they [the residents] meet their designated GP for the first time*” (SoHA2, care home 3).

Therefore, it was found better to introduce the PREPAIR-CH in a more stable phase when the resident had settled down e.g., in connection with the regular chronic care consultation (“It could be relevant to use in connection with such an annual review consultation [N1, care home 2]). Additionally, the HCPs suggested that the intervention was integrated into routine care for all settled residents.

##### Intervention refinement to the final model

Overall, the mechanisms of action in the proposed logic model (Fig. 2) were confirmed during testing. Based on the positive experiences, both GPs and staff supported to continue with the PREPAIR-CH and the medication communication template as key intervention components. To address the identified challenges, the alignment of expectations for involvement with resident and relatives was omitted, as specific training in this task was not considered feasible in a real-life setting with high staff turn-over. Additionally, the timepoint of the staff-led completion of the PREPAIR-CH was changed to being shortly before the GP consultation, at the discretion of the staff to ensure flexibility, instead of being conducted as part of the start-up meeting for newly arrived residents. Furthermore, the target group for the intervention was changed from newly arrived residents to settled residents and their relatives. With these changes, the intervention was adjusted to a simpler version with a more flexible workflow to better fit the local setting.

In alignment with the preliminary intervention model (Fig. 2), the final intervention included two fixed components (PREPAIR-CH and the medication communication template) which were to be delivered by the HCPs in a flexible three-stage workflow. Stage 1 included a staff-led completion of the PREPAIR-CH with the resident and, if possible, the relatives before the GP consultation. Stage 2 comprised the GP consultation, including a dialogue about the medication based on the PREPAIR-CH. Staff and relatives would participate and facilitate the dialogue if necessary and desired by the resident. Finally, stage 3 included follow-up after the GP consultation, where the medication communication template would be filled out by the GP and sent to the staff as part of the total treatment plan, if medication changes were agreed on and implemented.

#### Session 2: implementation strategy

With inspiration from the initial idea bank, the workshop participants discussed and selected relevant elements for the implementation strategy (Table 1) in session 2. The participants highlighted the following aspects as important: to inform the entire staff group about the project (“*present it to as many [staff] as possible”, [CM2, care home 2]*), to engage one or two coordinators at each care home, and to keep the informational materials simple and brief (“it has to be really short*”, [GP2, care home 3]*). The participants rejected instruction videos as implementation support.

## Discussion

### Main findings

The present paper describes the development of a complex intervention aiming to support person-centred medicine in the care home setting. We found that residents and relatives generally wished to be involved in medication-related decisions. Based on the resident and relative feedback, the original PREPAIR was modified to the PREPAIR-CH to better fit the care home population. Co-production workshops and testing with end-users guided the further development and refinement of the preliminary intervention drawn up in our programme theory. In this process, the intervention was adapted to fit the existing workflows and resources. The final complex intervention included two fixed components (PREPAIR-CH and the medication communication template) which were delivered through a flexible three-stage workflow. Additionally, a multi-component implementation strategy was developed.

### Comparison with existing literature

Several studies have demonstrated that care home residents and their relatives want to be involved in medication-related decisions [29, 56, 57], but many residents find it difficult [29, 58]. In line with our findings, previous research has shown that residents believe that the HCPs make the decisions about their medications and that they trust the HCPs decisions [29, 58, 59]. In our study, the HCPs confirmed this perception of usual care being mostly characterised by HCP decision-making.

We found that the intervention focus on resident and relative involvement was in alignment with the organisational and individual professional values articulated during the development process. Organisational values have been identified as an important influencing factor in the realisation of resident and relative involvement and shared decision-making [22, 60]. However, solely having a general focus on involvement is not sufficient to ensure actual involvement [22], and, in our study, the HCPs emphasised that involvement takes time and needs to be prioritized to occur more systematically.

A recent systematic review by Eidam et al. [61] identified 55 different tools that have been applied to evaluate patient preferences in geriatric pharmacotherapy. Only three tools targeted the context of multimorbidity-related polypharmacy, and none were found ideal for practicable elicitation of patient preferences in the context of geriatric polypharmacy. The main limitation of the tools was a time-consuming design. The review concluded that tools aiming to elicit patient preference should be simple and help to minimize the time investment in preference elicitation to meet the time constraints imposed by routine care [61].

The findings by Eidam et al. [61] aligns with a recent realist review from the International Patient Decision Aid Standards Collaboration. Based on data from 23 implementation studies, this review presented eight programme theories describing the mechanisms by which patient decisions aids become successfully implemented into routine health care settings [51]. According to these theories, intervention implementation is more likely to occur when the intervention contains simple tools that is integrated into the clinic workflow (which is often complex); when it prepares and prompts the patients to engage; and when a systematic delivery is used. These intervention characteristics are consistent with the intervention developed in our study. Importantly, implementing even a simple tool into real-life settings requires careful consideration of the context and existing pathways. This includes identification of the mechanisms that need to be changed and how to make these changes work in practice. We attempted this through thorough development based on programme theory and user involvement with coproduction and small-scale testing.

Our programme theory was based on combined knowledge from the PREPAIR study and the IP-SDM model. However, other theoretical and conceptual frameworks exist that have been used to support the development of patient involvement interventions [62]. An interesting theoretical framework for supporting complex intervention development and evaluation is the Making Informed Decisions Individually and Together (MIND-IT) [62]. Like the IP-SDM model, it represents explicitly the agency of multiple decision makers making the same healthcare decision from their different contexts. It also includes a central interaction point, where exchange of understanding, reasoning about preferences, and implementation of agreed choices takes place when sharing decision making in consultations. In contrast to the IP-SDM model, the MIND-IT outlines in greater detail various factors that can influence patient and HCP reasoning. This can be helpful to gain a deeper understanding of the active ingredients and mechanisms associated with multiple stakeholders´ reasoning and action. For instance, the MIND-IT highlights experience and skills as central influential factors. In our study, the performance of the intervention relied on the HCPs´ existing clinical experience and communication skills, as specific intervention training was considered unfeasible in a real-life care home setting. However, during testing, it became clear that the staff did not feel sufficiently prepared to perform the planned alignment of expectation, although this element was suggested by the staff in the co-producing workshop. These findings emphasize the importance of considering individual stakeholder factors, as they can have considerable impact on intervention feasibility and outcomes.

Overall, the intervention in our study was found to be feasible within the existing working routines and resources, except for the alignment of expectations which was omitted in the final model. The systematic delivery was found to disrupt the habits of usual care and increase HCP awareness about resident and relative involvement. The PREPAIR-CH was perceived to support dialogue and empower the residents to speak, thereby bringing new insights into the patient perspectives on their medications. Moreover, the medication communication template was perceived to be supportive for the staff during follow-up on medication changes. Hence, the mechanisms of actions suggested by our findings supported our programme theory. Furthermore, the final implementation strategy included multiple components aiming to facilitate whole-team engagement and knowledge, supportive leadership, and responsible implementation leaders in line with existing implementation theory [50] and evidence-based recommendations [51].

### Implications

This development study was conducted in accordance with the prevailing guidance on how to develop complex interventions drawing on a combination of approaches, including theory, existing evidence, and stakeholder partnership [35]. These approaches were applied flexibly to tailor the development process to our specific context. After the final refinement process, the developed intervention was perceived to be acceptable and feasible in the care home setting. The next step in our project is a feasibility study, in which the developed intervention and implementation strategy will be further tested, and key uncertainties will be explored.

A remaining key uncertainty is the role of relatives and how they perceive the intervention, as we were unable to recruit relatives in the testing of the intervention. Additionally, the most optimal timing of the intervention remains uncertain. The intervention initially targeted newly arrived residents; however, during testing, it was found more appropriate to include residents in a stable phase. Consequently, these aspects need further exploration in the feasibility study. Economic considerations are also a core element in the MRC framework, and the next phase will further explore the resource and outcome consequences of the intervention. The feasibility study will be conducted in two new care homes to strengthen the validity and generalisability of the research findings.

### Strengths and limitations

A major strength of this study was the combined use of theoretical, evidence-based, and practice-based knowledge to develop the intervention and implementation strategy. The MRC framework and the CFIR provided guidance on important aspects to consider during the development phase. Our programme theory drew on evidence-based work from the PREPAIR study and the IP-SDM model. Additionally, we included practice-based knowledge though stakeholder involvement, including relevant managerial and user levels. The use of co-production, rapid testing, and close collaboration with the municipality contributed to the development of a relevant and realistic intervention that fitted into the existing workflow at the care homes.

A limitation was that no residents or relatives participated in the workshops. As this was not considered feasible, we sought to include their perspectives though pre-workshop interviews and testing. Several activities were limited by the COVID-19 pandemic. Only few residents and no relatives participated in the testing, and observations were reduced to a minimum. This prevented us from gaining insights into the relatives**’** perspectives and may have limited the nuances to our findings. Likewise, the HCP perspectives were based on few, motivated individuals and might not generalise to other care homes. An inherent limitation to qualitative research also includes the researchers’ pre-understanding which may jeopardise the validity of study findings [63]. In this study, the development and presentation of a programme theory contributed to the illumination of the researchers**’** pre-understanding. Additionally, the use of co-producing processes and discussion of results in a cross-disciplinary research group helped to minimize the risk of unintended influence from pre-understanding.

## Conclusions

In this study, we developed a complex intervention aiming to support person-centred medicines in the care home setting through resident and relative involvement and interprofessional communication support. Presenting the details of the development process facilitate the transferability of this work and ensures that links can be made between the intervention development and the future success of the intervention or lack of such. The learnings of this development study suggest that the final intervention is acceptable and feasible for end-users. They further indicate that the intervention might be a viable approach to facilitate resident and relative involvement, which will be further explored in the planned feasibility study.

### Supplementary Information

Supplementary Material 1.

Supplementary Material 2.

## Data Availability

The data generated during the current study are not publicly available due to the sensitive nature of the data and personal information provided by participants. The data and other study materials are available from the corresponding author on reasonable request.

## Abbreviations

GP: General practitioner
HCP: Health care professional
MRC: Medical Research Council
PREPAIR: PREparing Patients for Active Involvement in medication Review
PREPAIR-CH: PREPAIR care home
rPATD: The Patients' Attitudes Towards Deprescribing
SoHA: Social and health care assistant
