# Supplementary material for: Person-centred medicine in the care home setting: development of a complex intervention
Source: BMC Prim Care. 2024 May 27;25:189. doi: 10.1186/s12875-024-02437-x (PMC11131350; doi:10.1186/s12875-024-02437-x)
Supplement: Supplementary file 1 — Supplementary Material 1. [file 12875_2024_2437_MOESM1_ESM.docx]

Supplementary material 1

**Interview guide: Care home residents/relatives**

|  | **Care home residents** | **Relatives** |
| --- | --- | --- |
| **THEME:** | Approx. 25-30 minutes | Approx. 15-20 minutes |
| **Background information** | 1. Could you tell me what it is like to live at the Thorsgaarden residential care home?   Supplementary (if applicable): For how long time have you lived here? Do you have visitors now and then? Who comes to visit you?   1. May I know your age? 2. Did you get a new doctor when moving to Thorsgaarden? What was that like? | 1. What is your relation to X? 2. Do you live close to the care home? Do you visit often? 3. May I know your age?   Supplementary (if applicable): What is your daily occupation? (if retired: What did you do when you were working?)   1. Do you have any concerns as a relative to X? |
| **Knowledge of/experience with own medications** | 1. Do you get any medications?      1. Do you know why you get your medications? Would you like to know? 2. How do you feel about taking your medications?   Supplementary (if applicable): How does taking medications affect your everyday life? Are you taking all your pills? | 1. Do you know which medications X gets? 2. Do you know what X gets the medications for? Would you like to know? |
| **Involvement in decisions on own medications** | 1. Who decides which medications you are to take? (yourself, doctor, relatives, care home staff, others?) 2. Do you feel involved in decisions on your medications? If not: Would you like to be? Optionally: Elaborate on how. 3. Who do you ask when in doubt about your medications? 4. What is important to you when talking to your own general practitioner about medications? |  |
| **Involvement of relatives in medication decisions** | 1. How do you want to be involved in your medications? Why? 2. Would you like your relatives to be involved in decisions about your medications? How? 3. Would you like the care home staff to be involved in decisions about your medications? How? | 1. Do you experience problems with your relative’s medications? 2. Do you believe that your relative experiences problems with his/her medications? 3. Do you feel involved in decisions on your relative’s medications? Would you like to be? How? 4. Who do you ask when in doubt about your relative’s medications?   Supplementary (if applicable): Do you have contact with X's general practitioner? |
| **Testing of the questionnaire “How do you feel about your medications?”**  (15 minutes) | The questionnaire should be tested in the presence of both the care home resident and the relative. | |
