# Supplementary material for: Person-centred medicine in the care home setting: development of a complex intervention
Source: BMC Prim Care. 2024 May 27;25:189. doi: 10.1186/s12875-024-02437-x (PMC11131350; doi:10.1186/s12875-024-02437-x)
Supplement: Supplementary file 2 — Supplementary Material 2. [file 12875_2024_2437_MOESM2_ESM.docx]

**Interview guide: Care home residents/relatives**

|  | **Care home residents** | **Relatives** |
| --- | --- | --- |
| **THEME:** |  |  |
| **Background** | 1. May I know your age? | 1. What is your relation to X? 2. Do you live close to the care home? Do you visit often? 3. May I know your age? 4. Supplementary (if applicable): What is your daily occupation? (if retired: What did you do when you were working?) |
| **Planning consultation** | 1. Did the care staff encourage you to ask questions or discuss worries regarding the medications at the following consultation with the doctor? |  |
| **Experienced involvement in medication consultation** | When you think about the last doctor's appointment you just had...   1. Did the doctor ask about your experiences with your medications? 2. Did you discuss questions or worries about the medications with the doctor? 3. Did the doctor make an effort to hear what matters most to you in relation to your medications? 4. Were you consulted when decisions were made about your medication? 5. Were efforts made to consider what is important to you in the decision about further medical treatment? (what will happen next in your treatment?) | Possibly same questions for relatives or let them participate in the conversation about experienced involvement. |
| **Questionnaire: How do you feel about your medication?** | 1. How did you fill in the questionnaire on your medications? 2. Alone 3. Together with relative 4. Together with care staff 5. Together with doctor 6. Did the questionnaire help you make decisions on your medical treatment? 7. How? 8. Did the questionnaire help you consider questions you would like to ask the doctor? 9. How? 10. Did the questionnaire prepare you for talking to your doctor about what matters most to you concerning your medications? 11. How?   These questions may already have been answered previously:   1. Can you tell me if (and how) the doctor asked about your own experiences with the medications? 2. Can you tell me if (and how) you were able to talk to the doctor about your questions or concerns about your medications? 3. Can you tell me if (and how) you were consulted when decisions were made about your medications? | Possibly same questions for relatives or let them participate in the conversation about the questionnaire. |
| **Own preference for involvement** | 1. Who decides which medications you should take? 2. You 3. Doctor(s) 4. Relates 5. Care staff 6. Others 7. Do you feel involved in decisions on your medications? 8. If not: Would you like to? Possibly elaborate on: How? 9. How do you prefer to be involved in decisions on your medications?  Support questions: 10. Would you prefer to make the final decision about your treatment yourself? 11. Would you prefer that you and your doctor share the responsibility of deciding which treatment is best for you? 12. Would you prefer to leave decisions about your treatment to your doctor? | Possibly same questions for relatives or let them participate in the conversation about involvement preference. |
| **Wrap-up and closing** | 1. Can you tell me what it was like to be part of the project? | 1. How did you experience being part of the project? 2. How did you experience X being part of the project? |
